# Extracting fetal heart signals from Doppler using semi-supervised convolutional neural networks

**DOI:** 10.3389/fphys.2024.1293328

**Published:** 2024-07-08

**Authors:** Yuta Hirono, Chiharu Kai, Akifumi Yoshida, Ikumi Sato, Naoki Kodama, Fumikage Uchida, Satoshi Kasai

**Affiliations:** ^1^ Major in Health and Welfare, Graduate School of Niigata University of Health and Welfare, Niigata, Japan; ^2^ TOITU Co. Ltd., Tokyo, Japan; ^3^ Department of Radiological Technology, Faculty of Medical Technology, Niigata University of Health and Welfare, Niigata, Japan; ^4^ Department of Nursing, Faculty of Nursing, Niigata University of Health and Welfare, Niigata, Japan

**Keywords:** Doppler, ultrasound, fetal heart signal, semi-supervised learning, deep learning

## Abstract

Cardiotocography (CTG) measurements are critical for assessing fetal wellbeing during monitoring, and accurate assessment requires well-traceable CTG signals. The current FHR calculation algorithm, based on autocorrelation to Doppler ultrasound (DUS) signals, often results in periods of loss owing to its inability to differentiate signals. We hypothesized that classifying DUS signals by type could be a solution and proposed that an artificial intelligence (AI)-based approach could be used for classification. However, limited studies have incorporated the use of AI for DUS signals because of the limited data availability. Therefore, this study focused on evaluating the effectiveness of semi-supervised learning in enhancing classification accuracy, even in limited datasets, for DUS signals. Data comprising fetal heartbeat, artifacts, and two other categories were created from non-stress tests and labor DUS signals. With labeled and unlabeled data totaling 9,600 and 48,000 data points, respectively, the semi-supervised learning model consistently outperformed the supervised learning model, achieving an average classification accuracy of 80.9%. The preliminary findings indicate that applying semi-supervised learning to the development of AI models using DUS signals can achieve high generalization accuracy and reduce the effort. This approach may enhance the quality of fetal monitoring.

## 1 Introduction

To mitigate the risk of fetal acidosis, it is crucial to assess fetal wellbeing during pregnancy, labor, and delivery. Cardiotocography (CTG) is widely used as a continuous monitoring method to assess fetal wellbeing. The International Federation of Gynecology and Obstetrics (FIGO) has provided guidelines on CTG interpretation, enabling obstetricians and midwives to analyze and evaluate fetal oxygenation based on CTG readings (Lewis et al., 2015). Signals for fetal heart rate (FHR) and uterine contractions in CTG correlate with the health status of the fetus (German Society of Gynecology and Obstetrics, 2014; Arnold and Gawrys, 2020). A well-traceable CTG, particularly with a continuous FHR that does not show any loss, can effectively reflect the fetal status.

The most common technology used to measure the FHR is based on Doppler ultrasound (DUS). The FHR is obtained from the average interval of the correlation coefficient peaks calculated using an autocorrelation function (ACF) applied to the DUS signals (Alnuaimi et al., 2017; Hamelmann et al., 2020). The DUS signal does not have a fixed amplitude peak for each heartbeat but occurs periodically, and the peak of the correlation coefficient is paired with the occurrence of a heartbeat (Marzbanrad et al., 2018). However, the FHR calculation algorithm with DUS and ACF has limitations and causes poor CTG recordings, such as signal loss and inaccurate FHR (e.g., double or half counts). Specialists frequently encounter CTGs with inadequate signal quality, which hinders their ability to diagnose fetal wellbeing, particularly during the second stage of labor (Nunes et al., 2014; Faiz et al., 2021; Reis-de-Carvalho et al., 2022). Mismatch between the signal range required for calculating correlation coefficients and the actual length of the obtained signals can result in double-counting or half-counting. This phenomenon occurs under conditions in which abnormal fetal heartbeats such as fetal arrhythmias or artifacts appear between beats. Signal loss occurs when the correlation coefficient peak interval is no longer paired with the FHR due to artifacts introduced by fetal or maternal motion within the DUS signal or due to the fetal heart moving out of the scan area (Marzbanrad et al., 2018; Hamelmann et al., 2020; Reis-de-Carvalho et al., 2022). Furthermore, if maternal blood vessels are present in the scan area, the heart rate is calculated from the beating of these blood vessels. This means that the fetal heart rate cannot be accurately determined and the signal related to the maternal heart rate can be calculated (Bakker et al., 2004; Lewis et al., 2015). During labor, the quality of FHR can deteriorate because of momentary noise interruptions. However, the signal-to-noise ratio representing the quality of DUS signals is sufficient for FHR calculation. The object size and distance considerably influence the quality, and as the gestational weeks progress, fetal heart enlargement and the distance between the fetus’ heart and the device decrease. Therefore, DUS signal quality can be excellent during delivery Hamelmann et al. (2019).

Several studies have addressed the limitations of DUS and ACF. Liang H. et al. (2022) investigated the efficacy of in-phase and quadrature demodulation in electronic fetal heart rate monitoring to reduce false reports of FHR doubling or halving, possibly due to fetal cardiac arrhythmias. Jezewski et al. (2011) used dynamic adjustments of the autocorrelation window, adaptive autocorrelation peak detection, and the determination of beat-to-beat intervals to reduce the number of erroneous cardiac cycle measurements. Hamelmann et al. (2019) used multiple transducer arrays to reduce the possibility of moving the fetal heart within the ultrasound range. Eliminating the artifact effect during FHR computation might be achievable through methods that either directly count fetal heartbeat onsets within the DUS or perform ACF by extracting only the waveform of the fetal heartbeat signal within the DUS. Both approaches necessitate the extraction of the fetal heartbeat segment from the DUS signal.

Among artificial intelligence (AI) technologies, deep learning (DL) has achieved remarkable success in various tasks, including classification (Krizhevsky et al., 2012). Because diverse types of data are processed by AI, it is widely applied in various research fields. Within obstetrics, some studies have directly assessed fetal hypoxia using CTG input and AI to determine whether the arterial cord blood pH level is below a certain threshold (Ogasawara et al., 2021; Liang Y. et al., 2022; Gude and Corns, 2022; Spairani et al., 2022; Liang and Lu, 2023). Other studies have investigated DL models that could determine whether the bpm displayed on CTG is maternal or fetal, which is not supported by existing CTG analysis systems (Signorini et al., 2003; Magawa et al., 2021; Boudet et al., 2022). In addition, some studies have also used DL models to filter maternal ECG signals and extract fetal ECG signals (Hasan et al., 2009; Fotiadou et al., 2021; Ghonchi and Abolghasemi, 2022; Mohebbian et al., 2023).

AI models typically require a large amount of training data to effectively learn intricate features within a dataset. For example, the MNIST dataset used for image recognition consists of labeled data with 60,000 samples for training and 10,000 samples for evaluation (El Kessab et al., 2013). Given the time-consuming nature of dataset creation, in particular labeling, utilizing prelabeled open data is a time-effective choice for developing AI. In obstetrics, while open data exists for CTG or fetal ECG, none exists for DUS (Marzbanrad et al., 2018). Consequently, creating training data using DUS requires considerable time and effort.

Semi-supervised learning has emerged as a strategy to alleviate the significant labeling effort associated with AI tasks (Miyato et al., 2019; Chen et al., 2020; Sohn et al., 2020). Semi-supervised learning improves the performance of AI generalization by using both labeled and unlabeled data for training. This approach streamlines the training data creation process by focusing on data containing features that require minimal labeling effort.

The objective of this study is to enhance CTG quality by accurately determining FHR in DUS signals without artifacts. We hypothesized that classifying DUS signals by type could be critical for CTG improvement. Identifying fetal heartbeats can prevent the use of artifacts in calculating FHR and reduce inaccuracies. We also focused on identifying single beats using DUS. By doing so, we aim to extract the parameters utilized to calculate the inter-beat interval from the corresponding occurrence times.

We propose using AI for classifying DUS signals into their respective signal types. To the best of our knowledge, AI is yet to be implemented for DUS signals. Thus, efficacy validation is necessary. A key challenge for the integration is the considerable costs associated with dataset creation. Therefore, this study confirms the effectiveness of semi-supervised learning in AI for classifying DUS signals.

## 2 Materials and methods

The study was conducted in accordance with the Declaration of Helsinki, and approved by the Institutional Review Board of the Niigata University of Health and Welfare (Approval No. 18890-220810).

### 2.1 Data acquisition

The research materials used in this study consisted of CTG data and simultaneously recorded DUS signals. All data used in this study were collected by TOITU Ltd. and shared with the investigators at Niigata University of Health and Welfare as anonymously processed information. A total of 528 datasets were used, comprising 442 cases with a non-stress test (NST) and 86 cases in the first or second stages of labor. The dataset included pregnant women aged 20 years and older, with gestational ages ranging from 24 to 41 weeks. There were no cases of twins or more, fetal or maternal deaths, necessitation for careful monitoring, cesarean sections and instrumental vaginal deliveries. The total recording times for NST and labor were 161 h, 23 min, and 34 s, and 218 h, 32 min, and 44 s, respectively. The CTG monitors used in this study were the MT-610 and MT-516 models (TOITU Ltd., Tokyo, Japan). These monitors transmit signals to the central server *via* Ethernet. The recorded data were obtained from the FHR and uterine contractions on the CTG. The FHR was sampled at a frequency of 4 Hz with a resolution of 0.25 bpm. Simultaneously, DUS signals were recorded at a frequency of 1 kHz with a 16-bit resolution using custom software.

### 2.2 Extraction of fixed data length for AI

During fetal monitoring, the DUS signal consists of three main components: the fetal heartbeat, artifacts, and low-level signals without any periodic pattern. Double counting, half counting, and signal loss typically occur when calculating FHR using ACF. These problems originate from malfunctions resulting from constraints on the signal length and the calculation of heartbeats even for signals other than the target beat.

A solution is to classify the DUS signals into respective categories for appropriate processing. We used four categories, that is, the three categorized previously mentioned components, in addition to our own defined categories. The first category is single fetal heartbeat, representing one cycle of heartbeat motion. The second category is artifact, representing transient signals due to various movements. The third category is low-level signal, representing signals with noise amplitude. The fourth category is multiple heartbeats, indicating the presence of two heartbeats within a segment. Detecting two beats within a specific interval in the categorization of DUS signals is crucial for recognizing the necessity of detecting heartbeats before and after them, which can prevent double counting and half counting. These categories were used as labels for the DUS signals to be classified by AI. The fetal heartbeat is the most important because its onset timing and duration are used to calculate indices, such as the FHR and heartbeat interval. When preparing the training data, the segment was based on this parameter and was adjusted to one beat length. Artifacts and low-level signals are factors that lead to loss in the existing FHR calculation system. The fourth category also led to half-counting in the system.

The categorized data underwent a semiautomated four-step process to reduce the cost of data preparation.

In the first process, we performed segmentation using filtering and thresholding, which are the same methodology as the ACF methods (Hamelmann, et al., 2020). To avoid mixing different beats, the segment length was set to 350 ms so that it is shorter than 400 ms, which was reported in a previous inter-beat interval evaluation (Jezewski et al., 2006). This process was specifically targeted at extracting single fetal heartbeats and includes artifacts that meet the requirements. We applied high- and low-pass filters to DUS to enhance the frequency components associated with fetal heart movement while suppressing noise. We extracted the segmented signal, which ranged from 250 ms to 350 ms, using a threshold-based approach. This threshold was dynamically updated at each fixed analysis interval, and a morphological closing process was applied to prevent the division of single fetal heartbeats (Haralick et al., 1987).

In the second process, we extracted amplitude signals below a threshold; these signals are considered noise in ACF methods (Hamelmann et al., 2020). The same filtering and segmentation methods as described above were used to extract noise levels with durations ranging from 250 ms to 350 ms.

In the third process, segments identified during the initial process were categorized into two groups, whereas signals extracted during the second were assigned to a single category. The classification used the ACF expressed in Eq. 1, where x is the DUS signal, n is the first sample of the autocorrelation window, N is the ACF window length, τ is the delay, and max is the delay at which the autocorrelation value is the maximum. In the ACF method, intervals in which a stable FHR can be derived correspond to pairs of beats and peaks of ACF output (Hamelmann, et al., 2020). Therefore, segments extracted in conjunction with *τ*_max_ were classified to be “Single fetal heartbeats”. By contrast, in cases in which the FHR could not be derived due to transient signal contamination, *τ*_max_ could not be obtained. Therefore, segments for which *τ*_max_ did not exist within the same interval were classified “Artifacts.” The data extracted in the second process with no *τ*_max_ in segments were classified as “low-level signals.”

In the fourth process, a novel segment was extracted from two individual fetal heartbeats to accommodate the presence of two beat fragments within the extracted data. The category of “multiple heartbeats” was created by shifting the extraction starting point of the first single fetal heartbeat by half the wavelength when two consecutive single fetal heartbeats were present. All the extracted data shorter than 350 ms were padded with zeros to achieve the fixed length.

**FIGURE 1:**
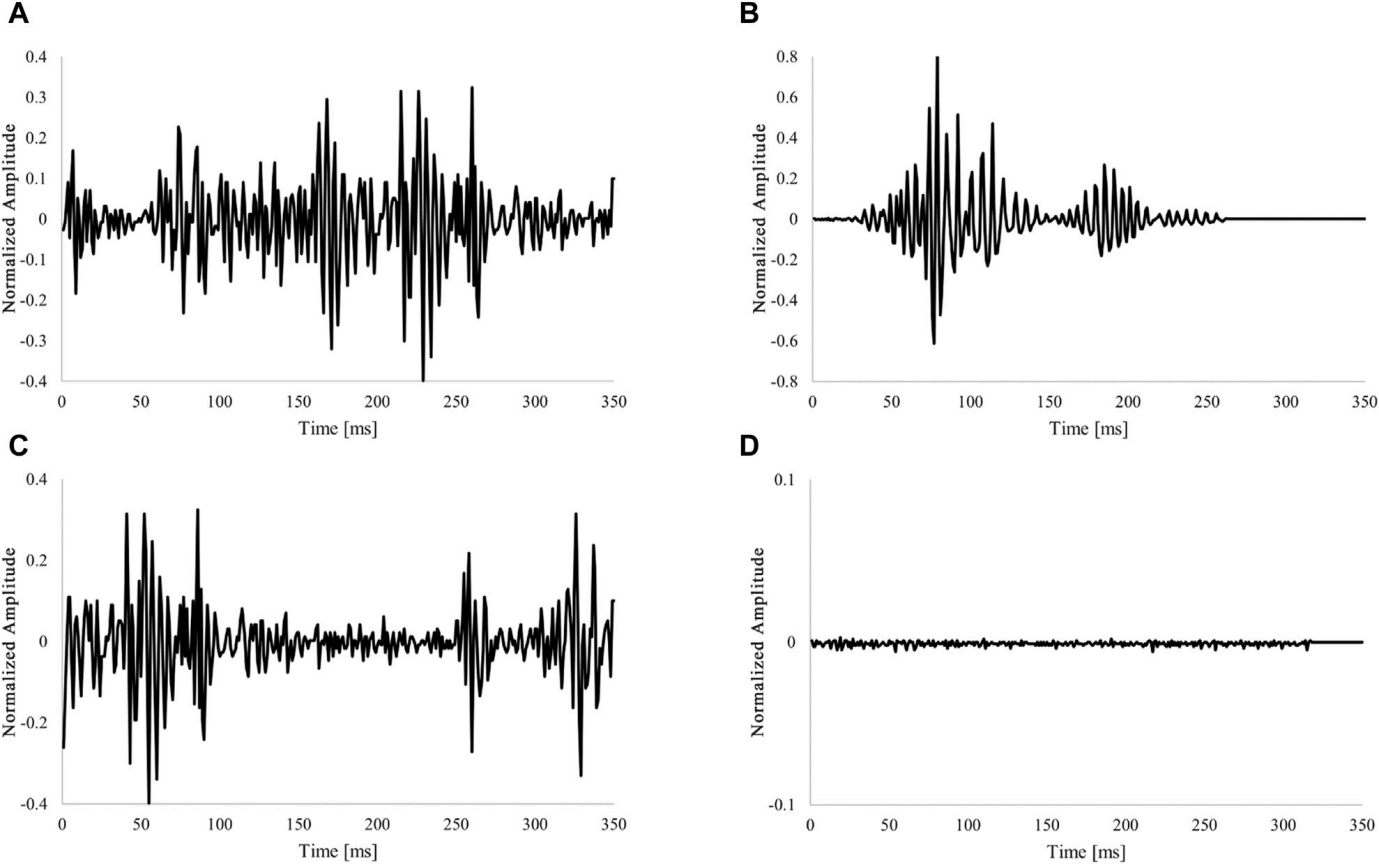
Example of labeling fixed-length data. **(A)** Waveform for one heartbeat (Single fetal heartbeat). **(B)** Non-periodic and transient signals (Artifacts). **(C)** Overlap with two heartbeat waveforms (multiple heartbeats). **(D)** Low amplitude and no characteristic behavior signal (Low-level signal). The amplitude of the acquired DUS signal was normalized to be within the 1 to 1 range.

As shown in Table 1, 602,409 data points were obtained and labeled for all categories. Examples of each extracted category are shown in Figure 1. Signal extraction and categorization algorithms were developed using the MATLAB R2022b software.

$$\text{ACF}\left[\tau \right]=\sum_{j=0}^{N-\tau }x\left(n+j\right)x\left(n+j+\tau \right),0\leq \tau \leq N$$ (1)

**TABLE 1:** Number of extracted data points in acquisition conditions.

Data label	Number of data points		
	Labor	NST	Labor + NST
Single fetal heartbeat	108,444	176,622	285,066
Artifact	9,045	8,762	17,807
Multiple heartbeats	108,444	176,622	285,066
Low-level signal	10,362	4,108	14,470

### 2.3 Data selection and dataset

Equal amounts of training data were used across all categories to develop an AI with high generalization performance. To investigate the effectiveness of semi-supervised learning, we required both labeled and unlabeled data. The results presented in Table 1 were generated automatically by the algorithm without researcher validation, acknowledging the impracticality of manually verifying each data point owing to time and cost constraints. Consequently, the researcher performed checks on the data to be labeled in the semi-supervised learning process. Recognizing the uncertainty surrounding the amount of training data required for DUS-based AI applications, a concerted effort was made to incorporate as much labeled data as possible. We visually inspected figures generated in units of several tens of seconds, which simultaneously depicted data generated by the semi-automatic labeling process and DUS signals, and confirmed that the generated labels matched the established definitions. The “labeled” data are the data that were checked by the researcher, and all other data were “unlabeled.”

The amount of data used in each category was adjusted to correspond to the category with the lowest count. The amount of labeled data was adjusted to approximately one-fifth that of the unlabeled data. The ratio of labeled to unlabeled data was set higher than that for other research with semi-supervised learning (Miyato et al., 2019; Sohn et al., 2020) to evaluate the accuracy by changing the number of labeled data points. A total of 2,400 labeled data points and 12,000 unlabeled data points were randomly selected from each category (Table 2). For training and validation purposes, 2,000 labeled data points were allocated, with 400 points for each category. Of the 9,600 labeled data points, 5,000 were from labor data and 4,600 were from NST data.

**TABLE 2:** Number of labeled and unlabeled data points for each category.

Data label	Number of data points		
	Labeled data		Unlabeled data
	Train	Validation	
Single fetal heartbeat	2,000	400	12,000
Artifact	2,000	400	12,000
Multiple heartbeats	2,000	400	12,000
Low-level signal	2,000	400	12,000

### 2.4 AI model

To create an AI model for classifying the four categories, we used a 1D-CNN for supervised learning. The core architecture comprises a series of three interconnected layers: convolutional, activation, and max-pooling layers. This sequence was repeated four times, and the probability of each option was calculated using a fully connected layer with softmax and classified as the option with the highest probability.

**FIGURE 2:**
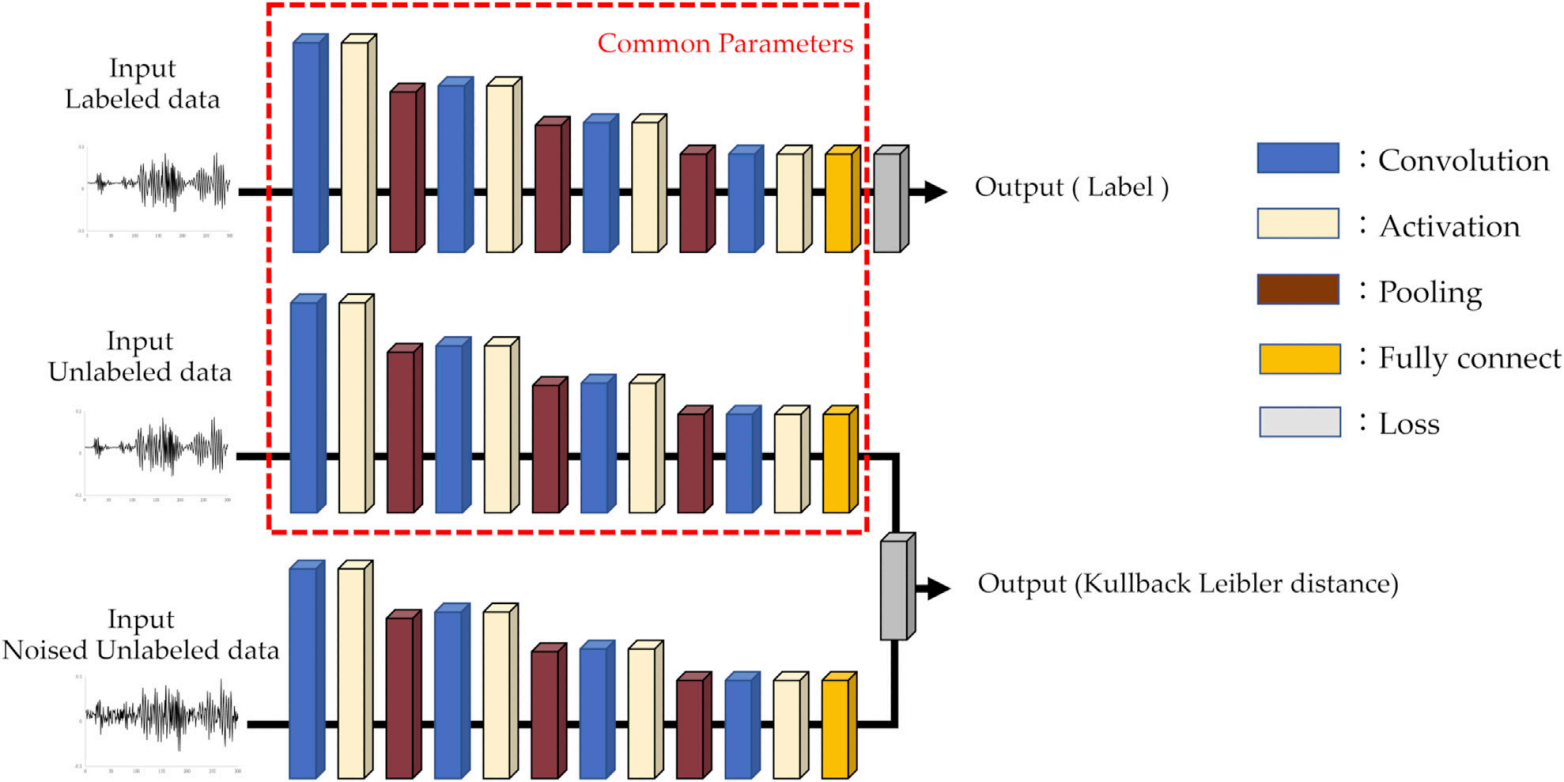
AI model architecture for semi-supervised learning.

The architecture of the AI model used for semi-supervised learning was identical to that of the supervised learning model, except for the loss function. The iterative process is as follows: First, supervised learning was performed on labeled data, followed by semi-supervised learning on unlabeled data. In the semi-supervised learning phase, both unlabeled data and data augmented with white noise were used. The noise-augmented data were added a random value to each point of the unlabeled data with an upper limit on the amplitude. The unlabeled data were processed in each layer using the same parameters used in the supervised learning. Similarly, unlabeled data with added noise were processed and trained to minimize the Kullback–Leibler distance. This iterative process is repeated. The AI model used in this study is illustrated in Figure 2. The AI was built using Sony Corporation’s Neural Network Console version 2.1. The specifications of the machine used for training and evaluation are as follows: An Intel(R) Core(TM) i9 series CPU operating at 3.00 GHz and an NVIDIA GeForce RTX 3090 GPU.

### 2.5 Performance evaluation

To evaluate the generalization performance of the AI, we performed a 6-fold cross-validation of the data listed in Table 2. To confirm the effectiveness of semi-supervised learning, we reduced the amount of training data in each fold to observe changes in accuracy. We randomly reduced the number of training data points to 8,000, 4,000, 2,000, 1,000, 500, and 240 while maintaining a consistent proportion for each category. Furthermore, the results of each 6-fold validation were combined and stratified based on the NST and labor data acquisition conditions to investigate whether any patterns of differences in accuracy were present.

Accuracy is often the most intuitive index used to evaluate model performance. However, in cases where data are unbalanced, accuracy may not be a reliable reference. Therefore, we also considered other indicators to evaluate the performance of the model in terms of precision, recall, and F-measure. The results can be classified into four categories: true negative (TN), false negative (FN), true positive (TP), and false positive (FP).

Accuracy indicates the proportion of samples that the model correctly predicts for the overall sample and assesses the overall model performance.

$$\text{Accuracy}=\frac{\text{TP}+\text{TN}}{\text{TP}+\text{FP}+\text{TN}+\text{FN}}\times 100\;\left[\%\right]$$ (2)

Precision indicates the proportion of samples that the model predicts as positive that are actually positive, and assesses the impact of false positives.

$$\text{Precision}=\frac{\text{TP}}{\text{TP}+\text{FP}}\times 100\;\left[\%\right]$$ (3)

Recall indicates the proportion of samples that the model correctly predicts as positive from the actual positive samples and assesses the impact of false negatives.

$$\text{Recall}=\frac{\text{TP}}{\text{TP}+\text{FN}}\times 100\;\left[\%\right]$$ (4)

F-Measure is the harmonic mean of the goodness-of-fit and reproducibility and provides a balanced assessment of the accuracy and sensing ability of the model.

$$F-\text{Measure}=\frac{2\times Precision\times Recall}{Precision+Recall}\times 100\;\left[\%\right]$$ (5)

## 3 Results

Table 3 presents the evaluation results for both supervised and semi-supervised learning, which are in the left and right halves of the table, respectively. The top and bottom halves of the table present the results for the training datasets of 240 and 8,000 data points, respectively. The precision, recall, and F-measures for each category are the average values derived from a 6-fold cross-validation.

**TABLE 3:** Performance of supervised and semi-supervised learning for each training data point.

Training data points		Supervised learning			Semi-supervised learning		
	Labels	Precision	Recall	F-measures	Precision	Recall	F-measures
240	Single fetal heartbeat	39.3	61.0	45.7	57.7	56.8	55.9
	Artifact	29.6	31.7	28.9	42.9	50.8	45.7
	Multiple heartbeats	61.8	47.0	51.4	80.3	69.9	74.6
	Low-level signal	51.0	28.9	34.5	96.0	84.3	88.9
8,000	Single fetal heartbeat	69.3	65.8	66.9	70.5	67.9	69.0
	Artifact	60.6	63.0	61.3	63.9	65.5	64.5
	Multiple heartbeats	91.9	92.8	92.3	92.4	93.0	92.7
	Low-level signal	97.9	97.0	97.4	97.0	97.0	97.0

In the supervised learning of the 240 training data points, the accuracy of each fold was in the range of 20.8%, from 32.3% to 53.1%, with an average accuracy of 42.1%. The highest average precision and F-measure were obtained for the multiple heartbeats at 61.8% and 51.4%, respectively. Single fetal heartbeat exhibited the highest average recall rate of 61.0%. In the semi-supervised learning of the 240 training data points, the accuracy of each fold was in the range of 18%, from 57.3% to 75.3%, with an average accuracy of 65.4%. The precision, recall, and F-measure had the highest averages for the low-level signal at 96.0%, 84.3%, and 88.9%, respectively.

In the supervised learning of the 8,000 training data points, the accuracy of each fold was in the range of 7.1%, from 75.3% to 82.4%, with an average accuracy of 79.6%; precision, recall, and F-measure all had the highest average for the low-level signal at 97.9%, 97.0%, and 97.4%, respectively. As shown by the results presented for semi-supervised learning on the 8,000 training data points, the accuracy of each fold was in the range of 4.1%, from 78.3% to 82.4%, with an average accuracy of 80.9%; precision, recall, and F-measure all had the highest averages for the low-level signal at 97.0%.

**FIGURE 3:**
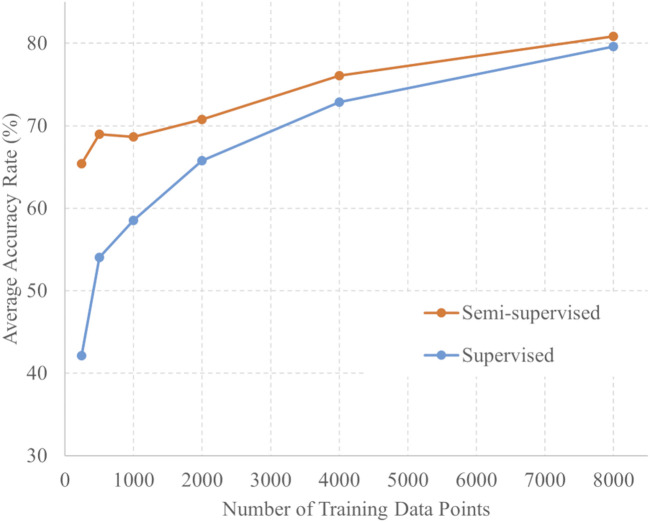
Number of training data points and accuracy of semi-supervised and supervised learning.

The number of training data points and average 6-fold accuracy for each training method are presented in Figure 3. For each amount of training data, the accuracy of semi-supervised learning consistently exceeded that of supervised learning. For each training data point, a *t*-test was conducted using the softmax outputs from both the supervised and semi-supervised learning. Across all training conditions, the *p*-values were less than 0.001 between supervised and semi-supervised learning. These statistical analyses demonstrate a significant difference between the two learning methods across all training data.

**FIGURE 4:**
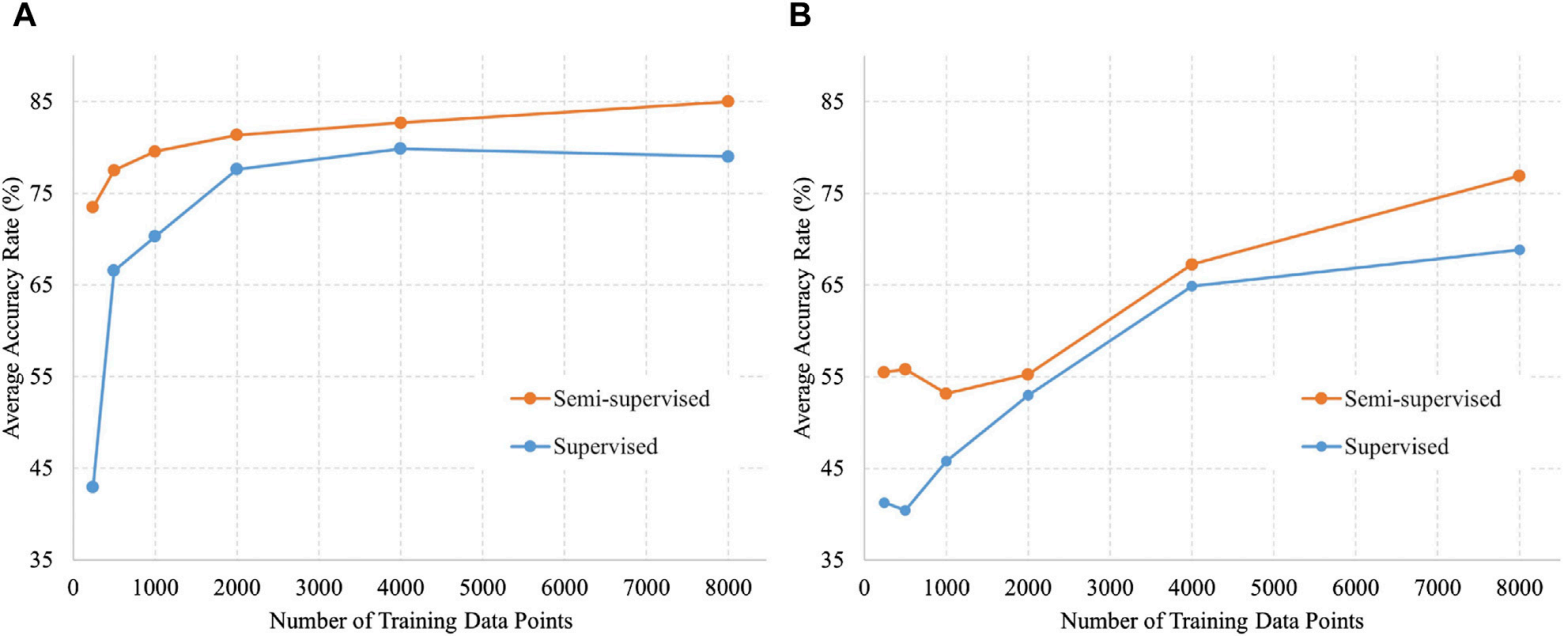
Number of training data and accuracy of semi-supervised and supervised learning for each data acquisition condition. **(A)** Data acquired by labor (5,000 data points); **(B)** Data acquired by NST (4,600 data points).

Figure 4 shows the validation data divided by acquisition conditions to identify trends. Figure 4A shows a comparison of accuracy against the number of training data in the labor dataset by the training method, and Figure 4B shows the same in the NST dataset. The trend in which the accuracy of semi-supervised learning was higher than that of supervised learning remained unchanged when the data were divided into different acquisition conditions. In addition, the accuracy appeared to be consistently higher for labor data compared with NST data.

In addition to the results presented here, Supplementary Table S1 also present the results from each fold for both semi-supervised and supervised learning, as well as the average accuracies and *p*-values depicted in Figure 3.

## 4 Discussion

Ultrasound-based FHR calculation methods are widely used in fetal monitoring systems. The DUS signal is used only for FHR calculations in conventional methods. However, the DUS signal inherently contains various factors necessary for the assessment of fetal cardiac function and a lot of potentially valuable information (Jezewski et al., 2006; Khandoker et al., 2009). We focused on DUS signals because it can provide useful information for fetal monitoring. In this study, the DUS signals were classified into four categories, including fetal heartbeats. To the best of our knowledge, this is the first study on DUS signals that applies AI to classify each type of signal during fetal heart rate monitoring.

The developed AI was tailored to classify four different waveforms of the observed DUS signals during fetal heart rate monitoring: single fetal heartbeat, artifact, low-level signal, and multiple heartbeats. Supervised learning resulted in an average accuracy of 79.6%, and semi-supervised learning achieved 80.9% accuracy. Previous studies involving AI for discriminating environmental sounds have presented models with discrimination accuracies ranging from 79.8% to 86.4%, which have led to successful classification outcomes (Su et al., 2019). Based on these results, it can be concluded that AI can be applied to DUS to extract crucial information such as fetal heartbeats and artifacts. This means that if DUS and its labels are prepared, various identifications are possible and may provide parameters that are not available in conventional fetal monitoring. An example is the distinction between maternal and fetal heart rates, which is difficult during fetal monitoring. This could lead to early attention in cases where the maternal heart rate is misidentified as the fetal heart rate during delivery (Pinto et al., 2015).

In Table 3, the results for single fetal heartbeat and artifact tend to be lower than those of other categories. Tables 4, 5 present the confusion matrices for each condition. Table 4 presents the confusion matrix for using 240 data points for learning, with a) representing semi-supervised learning and b) representing supervised learning. Table 5 presents the confusion matrix for using 8,000 data points for learning, with a) representing semi-supervised learning and b) representing supervised learning, respectively. These confusion matrices represent the combined results of all folds. In Tables 4, 5, the AI misclassified artifact as a single fetal heartbeat, and, except for Table 4 b), misclassified single fetal heartbeat as artifact. This phenomenon suggests similarities in the features of Artifact and Single fetal heartbeat. If this method is intended for medical use, misclassification can have fatal consequences. Therefore, further improvements in the accuracy are necessary. The accuracy could be improved by using additional parameters for training the AI, for example, the uterine contraction signal in the CTG. This may provide a potential guide for estimating the occurrence of artifacts. Additional training data should be required to separate these characteristics effectively. In addition, considering that this study achieved a classification accuracy of over 90% for overlapping heartbeat signals, combining multiple conditions to determine fetal heartbeats could improve the discriminative accuracy.

**TABLE 4:** Confusion matrix using 240 training data points.

(a) Semi-supervised learning					
		Predicted labels			
		Low-level signal	Single fetal heartbeat	Multiple heartbeats	Artifact
True Labels	Low-level signal	2,022	62	67	249
	Single fetal heartbeat	5	1,362	91	942
	Multiple heartbeats	47	132	1,677	544
	Artifact	51	859	271	1,219

(b) Supervised learning					
		Predicted labels			
		Low-level signal	Single fetal heartbeat	Multiple heartbeats	Artifact
True Labels	Low-level signal	693	573	195	939
	Single fetal heartbeat	50	1,465	244	641
	Multiple heartbeats	108	775	1,127	390
	Artifact	160	1,130	350	760

**TABLE 5:** Confusion matrix using 8,000 training data points.

(a) Semi-supervised learning					
		Predicted labels			
		Low-level signal	Single fetal heartbeat	Multiple heartbeats	Artifact
True Labels	Low-level signal	2,328	3	29	40
	Single fetal heartbeat	5	1,630	34	731
	Multiple heartbeats	31	26	2,232	111
	Artifact	37	670	121	1,572

(b) Supervised learning					
		Predicted labels			
		Low-level signal	Single fetal heartbeat	Multiple heartbeats	Artifact
True Labels	Low-level signal	2,328	0	26	46
	Single fetal heartbeat	2	1,579	28	791
	Multiple heartbeats	20	28	2,226	126
	Artifact	28	717	144	1,511

There are two potential developments from this study’s AI. The first is the classification in the pre-ACF phase. The FHR is calculated only when the DUS category, determined by using AI, is a single fetal heartbeat. Hence, the FHR could be improved by eliminating the effects of artifacts. The second is the direct FHR calculation by monitoring the timing of the occurrence of each fetal heartbeat. The FHR is obtained from the time interval between the characteristic cardiac activity signals (e.g., valve opening and closing) determined by AI within the DUS. The utilization of a specific cardiac activity signal, such as valve action, ensures robustness against fluctuations in the signal amplitude of the DUS, which affects the accuracy of the FHR in the ACF. Moreover, the FHR obtained through this approach could be closer to those obtained from ECG, which serves as the gold standard. Thus, this approach has the potential to provide an assessment of cardiac function by measuring not only FHR, but also the time of each cardiac function interval, such as short-term variability, whose parameters have generally only been measured using ECG with scalp electrodes (Gonçalves et al., 2006).

Semi-supervised learning has proven to be effective for tasks that require novel labeling, such as DUS signal labeling. Semi-supervised learning was found to reduce labeling costs and improve generalization performance, as confirmed in this study. The labeled data were validated by the researchers after algorithmic signal extraction and categorization. Employing semi-supervised learning has enabled researchers to reduce label-validation efforts by several dozen hours. In Table 3; Figures 3, 4, the accuracies of semi-supervised and supervised learning are higher than those of supervised learning. Furthermore, the variations in accuracy per fold in supervised learning were 20.8% for the 240 training data points and 18% for the 8,000 training data points. In contrast, the variations in accuracy per fold in semi-supervised learning were 7.1% for the 240 training data points and 4.1% for the 8,000 training data points. The variation in accuracy between each fold was smaller by approximately 3% in semi-supervised learning than in supervised learning. This confirms the robustness of semi-supervised learning. Therefore, the use of semi-supervised learning in DUS offers performance advantages.

**FIGURE 5:**
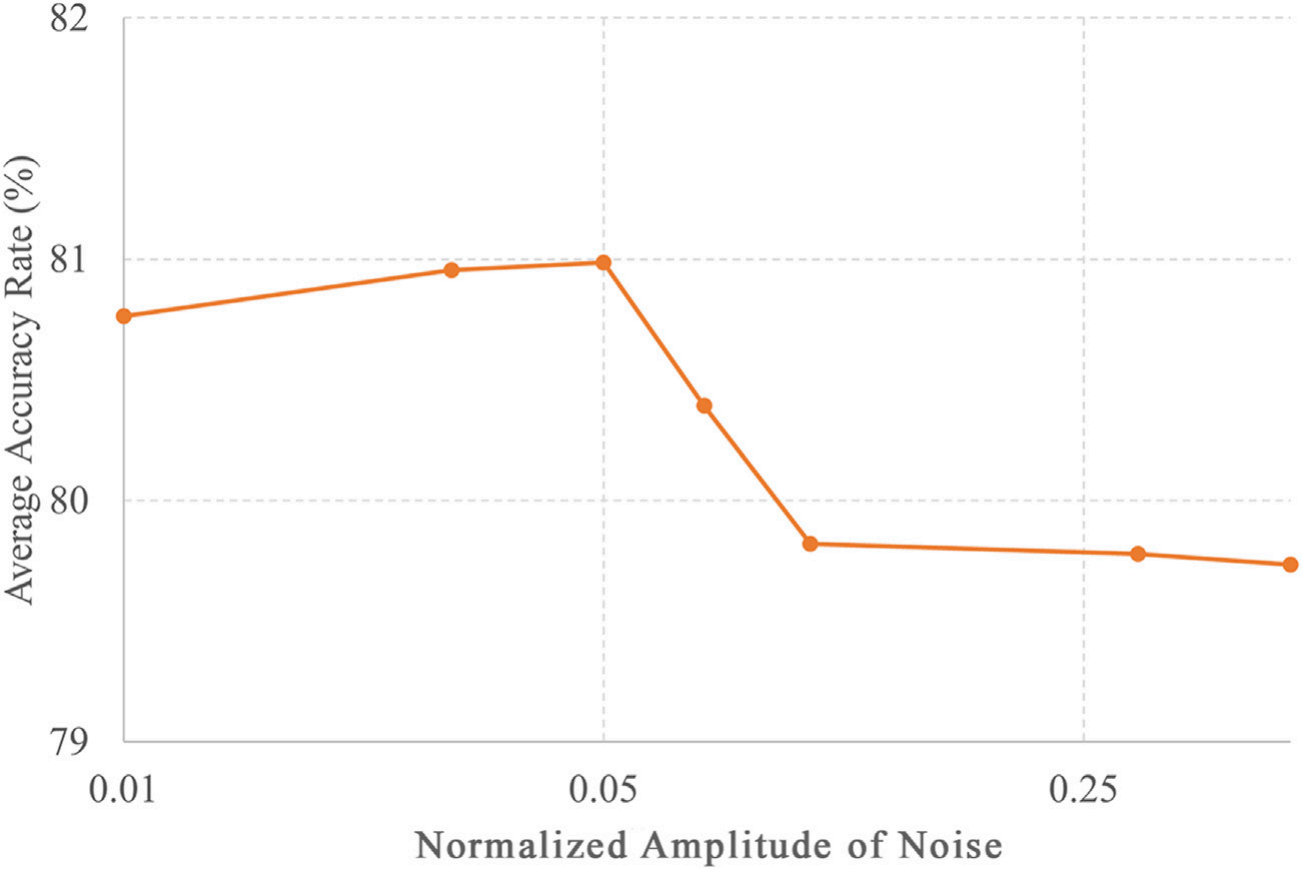
Noise amplitude and accuracy of unlabeled data in semi-supervised learning. The accuracies for different levels of noise amplitudes, including 0.01, 0.03, 0.05, 0.07, 1, 0.3, and 0.5, with the number of training data points set to 8,000.

In this study, the classification accuracy of each category of DUS was reported to be at most 80.9%, but there is potential for further improvement. This study employed a CNN and semi-supervised learning as the AI models. There are various types of AI models, such as ResNet (He et al., 2016), which extends the structure of CNNs, and Transformer (Vaswani et al., 2017), which exhibits high performance in natural language processing. The accuracy can be improved by using different models. Moreover, accuracy can be enhanced by choosing a particular method of semi-supervised learning. In this study, semi-supervised learning was used, which means that simple white noise was added to the unlabeled data, and the most accurate amplitude was selected based on the results shown in Figure 5. Nevertheless, various semi-supervised learning methods can improve accuracy. These methods include Virtual Adversarial Training (Miyato et al., 2019; Chen et al., 2020), which optimizes noise, and FixMatch (Sohn et al., 2020), which assigns pseudo-labels to improve generalization performance. Semi-supervised approaches can potentially enhance accuracy.

The results shown in Figure 4 suggest that the AI model developed in this study is more effective during labor. Given the higher likelihood of signal loss in CTG during labor, integrating this AI model during labor while retaining conventional methods during the NST could improve CTG quality. To improve the accuracy of the AI model in NST, further research is necessary to identify the factors that affect the difference in accuracy between labor and NST. Differences in fetal heart size may be an influential factor affecting accuracy. Because NST is conducted at an earlier gestational week than labor, it may result in a smaller fetal heart. The fetal heart size may have affected the signal-to-noise ratio (SNR) of the Doppler signal used in this study, owing to its correlation with the amplitude of the received ultrasound signal. Another contributing factor could be related to differences in the signal shape arising from variations in examination conditions. When the positions of the ultrasound probe and the fetal heart are not very close to each other, the fetal heart rate can still be calculated, even when the DUS signal is of poor quality, and the DUS signal may be more variable than during labor or delivery. NST is characterized by fewer uterine contractions and minimal movement by the mother and fetus, which Contraction h inherently reduces signal loss occurrences. In the cases examined in this study, the average signal loss was 4.6% during NST and 20.5% during labor.

The method used in this study to classify the DUS signals into multiple categories has the potential to accurately detect abnormal findings during fetal monitoring. For instance, it might be possible to diagnose fetal arrhythmias that were previously diagnosed using ultrasound diagnostic devices or fetal electrocardiograms using DUS signals for assessment (Jezewski et al., 2006; Khandoker et al., 2009; Wacker-Gussmann et al., 2014; Chivers et al., 2022). Table 3 shows that both supervised and semi-supervised learning methods tended to occasionally misclassify artifacts and fetal heartbeat components.

## 5 Limitations

In this study, we set the fixed data length to 350 ms for a preliminary investigation. This data length aims to prevent the overlap of two heartbeats and the splitting of one heartbeat. However, because there are some variations in each heartbeat, it is necessary to consider an AI model that can handle variable-length labeled data in the future (Fotiadou et al., 2021).

In addition, we used an algorithm to extract the fixed-length data and confirmed that the extracted waveforms matched each category, which was performed by an ultrasound engineer, because the confirmation was clear: waveforms within the extraction segment were compared based on the DUS shape, as indicated in previous studies (Jezewski et al., 2006; Khandoker et al., 2009).

Although categorization could be performed for the extracted fixed-length data, the algorithm used was strongly influenced by the SNR of the DUS signal, which resulted in heartbeats and artifacts that were not extracted.

We performed the classification in terms of three categories: fetal heartbeat signals that may occur during regular monitoring, artifacts, and no signals. However, one limitation is that we did not establish a framework to classify cases that deviate from normal fetal heartbeat signals, such as fetal arrhythmias. Fetal arrhythmias, which were not included in the fixed-length data used in this study, are known to produce longer or extremely short heartbeat waveforms. To classify abnormal fetal heartbeat waveforms, such as fetal arrhythmias, further investigation is required to develop DUS signal extraction methods for fetal arrhythmia findings and accrue a more extensive dataset.

## 6 Conclusion

To the best of our knowledge, this is the first study to explore the application of AI to DUS signals to improve fetal monitoring quality. We developed an AI model using semi-supervised learning to accurately classify DUS signals into four categories: single fetal heartbeat signals, artifacts, low-level signals, and multiple heartbeats.

The results of this study suggest that integrating semi-supervised learning into AI model development with DUS signals can efficiently lead to high generalization performance in terms of accuracy while simultaneously reducing the required labor-intensive efforts.

## Data Availability

The raw data supporting the conclusion of this article will be made available by the authors, without undue reservation.

## Abbreviations

ACF, Autocorrelation function; AI, Artificial intelligence; DL, Deep learning; DUS, Doppler ultrasound; FHR, Fetal heart rate; FIGO, Federation of Gynecology and Obstetrics; IQ, In-phase and quadrature; NST, Non-stress test; SNR, Signal-to-noise ratio; VAT, Virtual Adversarial Training.
