# Supplementary material for: Extracting fetal heart signals from Doppler using semi-supervised convolutional neural networks
Source: Front Physiol. 2024 Jul 8;15:1293328. doi: 10.3389/fphys.2024.1293328 (PMC11260753; doi:10.3389/fphys.2024.1293328)
Supplement: Supplementary file 1 [file Table1.DOCX]

Supplementary Material

# Supplementary Tables

In this study, we performed 6-fold cross validation to derive results for supervised learning and semi-supervised learning. Tables S1 and S2 present the results for 240 training data points, and Tables S3 and S4 present the results for 8,000 training data points. Table S5 presents the average accuracy and P-values for supervised and semi-supervised learning, respectively, with respect to the training data points.

**Supplementary Table S1.** Performance of supervised learning at 240 training data points.

|  |  | **Performance metrics %** | | | | | | |
| --- | --- | --- | --- | --- | --- | --- | --- | --- |
|  |  | **Fold1** | **Fold2** | **Fold3** | **Fold4** | **Fold5** | **Fold6** | **Average** |
| Single fetal heartbeat | Precision | 29.6 | 48.6 | 43.0 | 34.6 | 36.1 | 44.0 | 39.3 |
|  | Recall | 66.3 | 38.5 | 72.5 | 70.5 | 81.3 | 37.3 | 61.0 |
|  | F-Measures | 40.9 | 43.0 | 54.0 | 46.4 | 50.0 | 40.3 | 45.7 |
| Artifact | Precision | 25.9 | 22.4 | 33.2 | 26.8 | 38.0 | 31.3 | 29.6 |
|  | Recall | 25.8 | 52.3 | 26.3 | 18.8 | 22.3 | 44.8 | 31.7 |
|  | F-Measures | 25.8 | 31.4 | 29.3 | 22.1 | 28.1 | 36.8 | 28.9 |
| Multiple heartbeats | Precision | 50.2 | 70.8 | 69.2 | 60.5 | 74.7 | 45.3 | 61.8 |
|  | Recall | 37.3 | 59.5 | 56.8 | 30.3 | 31.8 | 66.3 | 47.0 |
|  | F-Measures | 42.8 | 64.7 | 62.4 | 40.3 | 44.6 | 53.8 | 51.4 |
| Low-level signal | Precision | 0.0 | 37.5 | 81.1 | 75.3 | 59.1 | 52.9 | 51.0 |
|  | Recall | 0.0 | 1.5 | 57.0 | 57.3 | 43.8 | 13.8 | 28.9 |
|  | F-Measures | 0.0 | 2.9 | 67.0 | 65.1 | 50.3 | 21.8 | 34.5 |
|  | Accuracy | 32.3 | 37.9 | 53.1 | 44.2 | 44.8 | 40.5 | 42.1 |

**Supplementary Table S2.** Performance of semi-supervised learning at 240 training data points.

|  |  | **Performance metrics %** | | | | | | |
| --- | --- | --- | --- | --- | --- | --- | --- | --- |
|  |  | **Fold1** | **Fold2** | **Fold3** | **Fold4** | **Fold5** | **Fold6** | **Average** |
| Single fetal heartbeat | Precision | 49.8 | 55.1 | 61.6 | 59.5 | 50.7 | 69.6 | 57.7 |
|  | Recall | 70.8 | 43.5 | 77.5 | 47.8 | 59.3 | 41.8 | 56.8 |
|  | F-Measures | 58.5 | 48.6 | 68.7 | 53.0 | 54.7 | 52.2 | 55.9 |
| Artifact | Precision | 47.2 | 41.4 | 58.9 | 42.8 | 26.9 | 39.9 | 42.9 |
|  | Recall | 44.5 | 53.3 | 46.3 | 60.0 | 36.8 | 64.0 | 50.8 |
|  | F-Measures | 45.8 | 46.6 | 51.8 | 49.9 | 31.0 | 49.2 | 45.7 |
| Multiple heartbeats | Precision | 87.5 | 82.8 | 83.5 | 85.1 | 84.1 | 58.8 | 80.3 |
|  | Recall | 71.8 | 62.8 | 81.0 | 73.0 | 71.3 | 59.5 | 69.9 |
|  | F-Measures | 78.8 | 71.4 | 82.2 | 78.6 | 77.1 | 59.1 | 74.6 |
| Low-level signal | Precision | 98.8 | 83.1 | 97.7 | 99.5 | 100.0 | 97.1 | 96.0 |
|  | Recall | 80.8 | 97.0 | 96.5 | 93.3 | 61.8 | 76.3 | 84.3 |
|  | F-Measures | 88.9 | 89.5 | 97.1 | 96.3 | 76.4 | 85.4 | 88.9 |
|  | Accuracy | 66.9 | 64.1 | 75.3 | 68.5 | 57.3 | 60.4 | 65.4 |

**Supplementary Table S3.** Performance of supervised learning at 8,000 training data points.

|  |  | **Performance metrics %** | | | | | | |
| --- | --- | --- | --- | --- | --- | --- | --- | --- |
|  |  | **Fold1** | **Fold2** | **Fold3** | **Fold4** | **Fold5** | **Fold6** | **Average** |
| Single fetal heartbeat | Precision | 71.1 | 70.6 | 79.2 | 75.7 | 61.3 | 58.0 | 69.3 |
|  | Recall | 70.0 | 59.5 | 56.0 | 66.3 | 71.3 | 71.8 | 65.8 |
|  | F-Measures | 70.5 | 64.6 | 65.6 | 70.7 | 65.9 | 64.1 | 66.9 |
| Artifact | Precision | 63.7 | 59.8 | 62.6 | 64.6 | 58.4 | 54.7 | 60.6 |
|  | Recall | 66.3 | 71.8 | 77.0 | 72.0 | 51.5 | 39.3 | 63.0 |
|  | F-Measures | 64.9 | 65.2 | 69.1 | 68.1 | 54.7 | 45.7 | 61.3 |
| Multiple heartbeats | Precision | 93.3 | 94.5 | 89.9 | 92.8 | 92.3 | 88.5 | 91.9 |
|  | Recall | 90.8 | 90.8 | 93.8 | 93.8 | 93.0 | 94.5 | 92.8 |
|  | F-Measures | 92.0 | 92.6 | 91.8 | 93.3 | 92.6 | 91.4 | 92.3 |
| Low-level signal | Precision | 96.5 | 99.0 | 96.8 | 97.5 | 100.0 | 97.7 | 97.9 |
|  | Recall | 96.8 | 98.8 | 98.8 | 97.5 | 94.8 | 95.5 | 97.0 |
|  | F-Measures | 96.6 | 98.9 | 97.8 | 97.5 | 97.3 | 96.6 | 97.4 |
|  | Accuracy | 80.9 | 80.2 | 81.3 | 82.4 | 77.6 | 75.3 | 79.6 |

**Supplementary Table S4.** Performance of semi-supervised learning at 8,000 training data points.

|  |  | **Performance metrics %** | | | | | | |
| --- | --- | --- | --- | --- | --- | --- | --- | --- |
|  |  | **Fold1** | **Fold2** | **Fold3** | **Fold4** | **Fold5** | **Fold6** | **Average** |
| Single fetal heartbeat | Precision | 70.5 | 76.2 | 76.8 | 73.4 | 63.5 | 62.8 | 70.5 |
|  | Recall | 72.8 | 65.0 | 63.0 | 70.5 | 69.3 | 67.0 | 67.9 |
|  | F-Measures | 71.6 | 70.2 | 69.2 | 71.9 | 66.3 | 64.8 | 69.0 |
| Artifact | Precision | 66.3 | 65.2 | 64.9 | 66.3 | 60.9 | 59.7 | 63.9 |
|  | Recall | 65.5 | 74.0 | 75.5 | 67.5 | 55.3 | 55.3 | 65.5 |
|  | F-Measures | 65.9 | 69.3 | 69.8 | 66.9 | 57.9 | 57.4 | 64.5 |
| Multiple heartbeats | Precision | 92.9 | 92.0 | 92.6 | 92.2 | 92.6 | 91.9 | 92.4 |
|  | Recall | 92.3 | 92.5 | 91.3 | 91.8 | 94.5 | 95.8 | 93.0 |
|  | F-Measures | 92.6 | 92.3 | 91.9 | 92.0 | 93.6 | 93.8 | 92.7 |
| Low-level signal | Precision | 97.7 | 97.5 | 95.2 | 95.9 | 97.2 | 98.4 | 97.0 |
|  | Recall | 96.5 | 98.3 | 98.3 | 98.5 | 95.5 | 95.0 | 97.0 |
|  | F-Measures | 97.1 | 97.9 | 96.7 | 97.2 | 96.3 | 96.7 | 97.0 |
|  | Accuracy | 81.8 | 82.4 | 82.0 | 82.1 | 78.6 | 78.3 | 80.9 |

**Supplementary Table S5.** Number of training data points, accuracy of semi-supervised and supervised learning, and p-values from t-tests.

|  | **Average Accuracy rate %** | |  |
| --- | --- | --- | --- |
| **Number of training data Points** | **Supervised** | **Semi-supervised** | **P-value** |
| 240 | 42.1 | 65.4 | < 0.001 |
| 500 | 54.0 | 69.0 | < 0.001 |
| 1,000 | 58.5 | 68.7 | < 0.001 |
| 2,000 | 65.8 | 70.8 | < 0.001 |
| 4,000 | 72.9 | 76.1 | < 0.001 |
| 8,000 | 79.6 | 80.9 | < 0.001 |
